# Hemolytic disease of the fetus and newborn: rapid review of postnatal care and outcomes

**DOI:** 10.1186/s12884-023-06061-y

**Published:** 2023-10-18

**Authors:** Derek P. de Winter, Allysen Kaminski, May Lee Tjoa, Dick Oepkes, Enrico Lopriore

**Affiliations:** 1 Department of Pediatrics, Division of Neonatology, Willem-Alexander Children’s Hospital, Leiden University Medical Center, Leiden, The Netherlands; 2 Department of Immunohematology Diagnostic Services, Sanquin Diagnostic Services, Amsterdam, The Netherlands; 3 OPEN Health, Bethesda, MD, USA (Currently The George Washington University, Washington, DC USA; 4 Janssen Pharmaceuticals, Raritan, NJ USA; 5 Division of Fetal Medicine, Department of Obstetrics, Leiden University Medical Center, Leiden, The Netherlands

**Keywords:** Hemolytic disease of the fetus and newborn (HDFN), Postnatal treatment, Postnatal outcomes

## Abstract

**Background:**

Advances in postnatal care for hemolytic disease of the fetus and newborn (HDFN) have occurred over the past decades, but little is known regarding the frequency of postnatal treatment and the clinical outcomes of affected neonates. Most studies reporting on HDFN originate from high-income countries or relatively large centers, but important differences between centers and countries may exist due to differences in prevalence and available treatment options. We therefore aimed to evaluate the postnatal treatment landscape and clinical outcomes in neonates with Rhesus factor D (Rh(D))- and/or K-mediated HDFN and to provide recommendations for future research.

**Methods:**

We conducted a rapid literature review of case reports and series, observational retrospective and prospective cohort studies, and trials describing pregnancies or children affected by Rh(D)- or K-mediated HDFN published between 2005 and 2021. Information relevant to the treatment of HDFN and clinical outcomes was extracted. Medline, ClinicalTrials.gov and EMBASE were searched for relevant studies by two independent reviewers through title/abstract and full-text screening. Two independent reviewers extracted data and assessed methodological quality of included studies.

**Results:**

Forty-three studies reporting postnatal data were included. The median frequency of exchange transfusions was 6.0% [interquartile range (IQR): 0.0–20.0] in K-mediated HDFN and 26.5% [IQR: 18.0–42.9] in Rh(D)-mediated HDFN. The median use of simple red blood cell transfusions in K-mediated HDFN was 50.0% [IQR: 25.0–56.0] and 60.0% [IQR: 20.0–72.0] in Rh(D)-mediated HDFN. Large differences in transfusion rates were found between centers. Neonatal mortality amongst cases treated with intrauterine transfusion(s) was 1.2% [IQR: 0–4.4]. Guidelines and thresholds for exchange transfusions and simple RBC transfusions were reported in 50% of studies.

**Conclusion:**

Most included studies were from middle- to high-income countries. No studies with a higher level of evidence from centers in low-income countries were available. We noted a shortage and inconsistency in the reporting of relevant data and provide recommendations for future reports. Although large variations between studies was found and information was often missing, analysis showed that the postnatal burden of HDFN, including need for neonatal interventions, remains high.

**Systematic review registration:**

PROSPERO 2021 CRD42021234940. Available from: https://www.crd.york.ac.uk/prospero/display_record.php?ID=CRD42021234940.

**Supplementary Information:**

The online version contains supplementary material available at 10.1186/s12884-023-06061-y.

## Background

In neonates affected by hemolytic disease of the fetus and newborn (HDFN), maternally formed immunoglobulin G (IgG) antibodies directed against the child’s erythrocytes invoke a persistent hemolysis. In recent decades, advances in the identification, care, and management of these affected infants have been developed and improved. The prevalence of Rhesus D (Rh(D))-mediated HDFN has significantly decreased due to the introduction of Rh(D) immunoprophylaxis (RhIG) [1–4]. Also, cases at risk of mild to severe hemolysis can now be detected and monitored antenatally with serological parameters [5–8] and ultrasound, [9–11] and treated with intrauterine transfusions in cases of severe fetal anemia. However, the prevalence of Rh(D)-mediated HDFN, the availability of RhIG and prenatal and postnatal treatment options may vary across countries due to sociodemographic, political, economic and geographical differences [12].

Postnatal treatment of HDFN focuses on preventing and minimizing the potential complications of anemia and hyperbilirubinemia [13]. Potential permanent consequences of severe hyperbilirubinemia, such as bilirubin encephalopathy, are prevented by intensive phototherapy and exchange transfusions. Hemoglobulin levels and reticulocyte counts may be monitored throughout the first 3 months of life, and anemia may treated with simple red blood cell (RBC) transfusions if needed. Currently, there are no approved therapies for HDFN.

Even though advances have been made, much is still unknown on the exact frequency of the need for postnatal treatment and the clinical outcomes of these affected neonates. Previous studies have reported on the rate of thrombocytopenia [14], iron overload [15], cholestasis [16–19], necrotizing enterocolitis [20] and sepsis [21] in HDFN-affected neonates, although many of these studies originate from the national referral center for fetal therapy in the Netherlands and other centers from high-income countries and may therefore not be directly translatable to other centers and countries. Large differences between centers and countries in, for instance, the rate of exchange transfusions, RBC transfusions and associated morbidities may exist, but have not been addressed before. Therefore, this rapid review aims to gain an insight into the postnatal treatment landscape, clinical outcomes, and burden of the disease in neonates affected by Rh(D)- and/or Kell (K)-mediated HDFN and to identify gaps in the currently available literature and subsequently provide recommendations for future research.

## Methods

### Literature searches

Research questions were devised using a PICO structure (Supplementary Table 1).The protocol for this study was developed according to Preferred Reporting Items for Systematic Review and Meta-Analysis (PRISMA) guidelines [22] and MOOSE Reporting Guidelines for Meta-Analysis of Observational Studies [23] and registered prospectively on PROSPERO (CRD42021234940). Medline, ClinicalTrials.gov and EMBASE were searched for journal articles and congress abstracts (EMBASE only) published between January 1, 2005 and March 10, 2021. A restriction on publication date was set due to improvements in intensive phototherapy and its application throughout the decades, possibly resulting in a decreased role for exchange transfusions in the management of hyperbilirubinemia. The search was conducted using ProQuest (Appendix S1). The search strategy and used search terms are also defined in the prenatal companion manuscript [24]. Studies reporting on postnatal treatment and clinical outcomes were included in this manuscript. No restrictions were set on studies reporting on cases before January 1, 2005. Duplicate articles were removed automatically. Additionally, reference lists from relevant systematic literature reviews of cohort studies were hand-searched, together with the authors’ personal libraries.

### Study selection

Two independent reviewers (D.P.d.W. and A.K.) reviewed citations, firstly the titles/abstracts in Rayyan (https://rayyan.ai/cite) and then the full texts, which were extracted into Microsoft Excel. Both reviewers hold a Masters degree relevant to the topic. Project director (D.O.) and the project team adjudicated decisions. Observational studies, trials, modeling studies, systematic reviews of cohort studies (to identify primary studies only), and case reports and case series of infants or children experiencing or having experienced Rh(D)- or K-mediated HDFN were included (Appendix S2). Non–English-language articles were excluded, as were notes, editorials, and commentaries; nonsystematic reviews; reports of populations, interventions, outcomes, or study designs not of interest; publication types not of interest; indexed conference abstracts; and reports of animal or preclinical studies.

Two reviewers (D.P.d.W. and A.K.) extracted data independently for each study (presented in Supplementary Table 2), including study characteristics (i.e., citation information, study design, and data source), patient characteristics (i.e., population description, sample size, antigen status), treatment patterns (i.e., use of phototherapy, exchange transfusion, simple RBC transfusion, or other treatments), and clinical outcomes (i.e., mortality, anemia, hyperbilirubinemia). Additionally, reporting of guidelines and thresholds used for exchange transfusions and simple RBC transfusions were collected. Discrepancies in data extraction between reviewers were resolved and the project director (D.O) adjudicated decisions in data extraction. Extracted variables were predefined by the authors. The Population, Intervention, Comparison, Outcomes, and Study (PICOS) design was used as a framework to formulate the study eligibility criteria [25]. Abstracted data underwent quality control by the project director (D.O.) who screened 10% of included/excluded articles. Methodological quality of the selected studies was independently assessed (D.P.d.W. and A.K.) using the JBI Critical Appraisal Checklist for Case Reports and Case Series, Newcastle–Ottawa Scale for observational studies, Checklist for Reporting Results of Internet E-Surveys (CHERRIES) for questionnaires and the NICE-checklist for randomized controlled trials.

### Analyses

Data from eligible studies were characterized as representative, which included data from studies that accurately reflected the characteristics of the larger group (e.g., larger case series, retrospective or prospective studies, randomized controlled trials [RCTs]), or nonrepresentative, which included data from studies that reflected a small proportion of the characteristics of the larger group (e.g., case reports or small case series, or studies in a subset of the larger group, such as cases treated with intrauterine transfusion, or cases with hydrops fetalis). When possible, we aggregated information reported in a similar manner. For unique outcomes, we highlighted information from generalizable studies. Where appropriate, data were summarized as percentage (mean ± standard deviation [SD] or range) or median (interquartile range [IQR]) for patient groups or patient populations.

Assessment of available findings was conducted to identify evidence gaps, and recommendations to fill unmet needs were formulated. Results pertinent to neonates are reported here; maternal and fetal outcomes and methods as described above have been reported in a companion publication by de Winter et al. [24].

Methodological quality was assessed by two independent reviewers (D.P.d.W. and A.K) until consensus was reached, using the JBI Critical Appraisal Checklist for Case Reports, JBI Critical Appraisal Checklist for Case Series, Newcastle–Ottawa Scale for retrospective and prospective cohort studies, Checklist for Reporting Results of Internet E-Surveys (CHERRIES) for questionnaires and the NICE Checklist for randomized controlled trials. The overall methodological quality scores are reported in Supplementary Table 6. Detailed methodological analyses are available in the prenatal companion publication [24].

## Results

### Study selection and characteristics

**Fig. 1:**
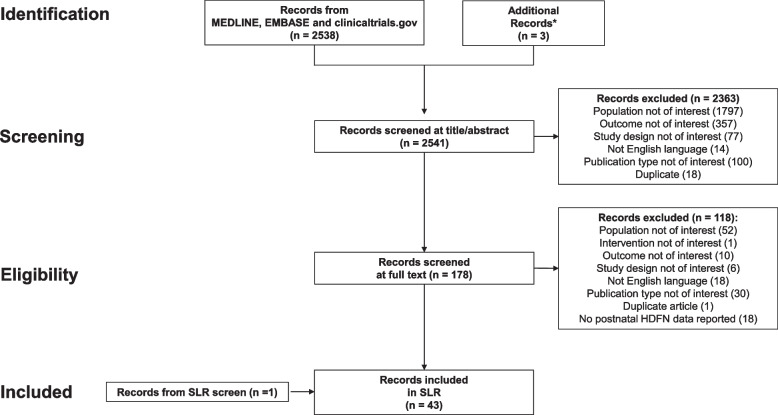
PRISMA flow diagram of study selection. SLR, systematic literature review. ^*^From authors’ personal libraries

A total of 2538 articles were identified through searches on Medline, ClinicalTrials.gov and EMBASE, and an additional 3 articles were identified from personal libraries (Fig. 1). Initially, 2363 articles were excluded based on the title and abstract screening and 136 articles were excluded based on full-text review. An additional article was identified through a reference search from eligible systematic reviews and cohort studies, resulting in a total of 60 included studies. Finally, 43 out of 60 studies reported postnatal data. Characteristics of the 43 included studies are reported in Supplementary Table 2 [16, 20, 26–66].

The included studies were comprised of 12 (28%) case reports, 6 (14%) case series, 1 (2%) questionnaire, 19 (44%) retrospective cohort studies, 4 (9%) prospective cohort studies, and 1 (2%) RCT. Studies were performed in 22 countries, most originated from Turkey (*n* = 6 [14%]), followed by The Netherlands (*n* = 5 [12%]) and the United States (*n* = 5 [12%]).

The 43 studies comprised 113 groups of neonates/infants, of which 12 (11%) were from single case reports and 14 (12%) were from case series (Table 1). The selected studies reported on cases managed between 1985 and 2019. Mean (range) group size was 36.5 (1–235). Mean (SD) gestational age was 35.0 (32.3–40.2) weeks, and mean birth weight was 2.67 (2.22–3.19) kg.

**Table 1:** Patient characteristics among neonates and infants from 43 eligible studies

Characteristic	Mean (range)	Patient groups (n)	No. of studies
Patient group size, n	36.5 (1–235)	113	43
Gestational age at birth, weeks			
Mean	35.0 (32.3–40.2)	11	7
Median	35.8 (32.0–37.0)	19	9
Exact	34.2 (28.1–38.1)	16	12
Birth weight, grams			
Mean	2670 (2220–3190)	11	7
Exact	2346 (1385–3750)	20	13

### Methodological quality of the studies

Six of 12 included case reports received a perfect score, whereas the remaining six studies lacked in accurately reporting the patients previous history, current condition or post-intervention condition (Supplementary Table 6). Only one case series received a perfect score, the remaining six case series lacked in accurately reporting the inclusion process and the clinical outcomes and follow-up. Of the 20 retrospective studies, 18 were of good quality and 2 of fair quality. All prospective studies were of good quality. The methodological quality of these studies were also described in more detail in the prenatal study of the same literature search [24].

### Treatment landscape

Data on intensive phototherapy was available in 23 studies (Supplementary Table 3,) of which 15 studies provided representative data with a median use of 99% [IQR: 75.7–100.0] for Rh(D)-mediated HDFN and 95.5% [IQR: 88.3–100.0] for K-mediated HDFN (Table 2). No adverse events related to intensive phototherapy were described. Use of exchange transfusions was reported in 27 studies. Eighteen studies reported representative data on the frequency of exchange transfusions. In these studies, the frequency of exchange transfusions was found to be lower in K-mediated HDFN (median use of 6.0% [IQR: 0.0–20.0]) in comparison to Rh(D)-mediated HDFN (median use of 26.5% [IQR: 18.0–42.9]). The frequency of simple RBC transfusions was reported in 19 studies, of which 11 provided representative data. Median use of simple RBC transfusions in Rh(D)-mediated HDFN was 60.0% [IQR: 20.0–72.0] and 50.0% [IQR: 25.0–56.0] in K-mediated HDFN. We were unable to accurately extract adverse events related to exchange transfusions or simple RBC transfusions from the included articles. Two studies reported on the use of intravenous immunoglobulin (IVIG) in Rh(D) or K-mediated HDFN [32, 40]. One retrospective study reported on the employment of delayed cord clamping [52]. A single case report reported on the use of recombinant erythropoietin in K-mediated HDFN [45].

**Table 2:** Frequencies of treatments employed in Rh(D)- and K-mediated HDFN

Treatment type^a^	Rh(D)	Kell
Intensive phototherapy	**99 [75.7–100]**	**95.5 [88.3–100]**
Exchange transfusion	**26.5 [18–42.9]**	**6.0 [0–20]**
Simple RBC transfusion	**60 [20-72]**	**50 [25-56**]

^a^Data presented as median % [IQR]

### Neonatal outcomes

Various adverse events were reported in 7 studies, of which 4 studies provided representative data (Supplementary Table 4). We were unable to aggregate the data due to a large heterogeneity in patient characteristics and employed treatment types. No studies reported representative data on cardiac dysfunction, necrotizing enterocolitis, the use of (non-)mechanical ventilation, or the use of umbilical venous catheters. Neurodevelopmental outcome was reported by 3 studies, where 25% (2/8) of patients presented with neurodevelopmental abnormalities. The case series by Harper 2006 reported on the neurodevelopmental outcome in a cohort of 16 post-hydropic children who were treated with an intrauterine transfusion for HDFN. Five cases, 3 with Rh(D)-mediated HDFN and 2 with K-mediated HDFN were reported upon and included in this review (Supplementary Table 4). Twelve studies reported on neonatal mortality rates (Supplementary Table 5). The median neonatal mortality rate among neonates treated with intrauterine transfusion(s), reported in 5 studies, was 1.2% [IQR: 0–4.4]. Aggregation of neonatal mortality rates for other included cohorts was not possible due to large variations in employed treatment options.

### Reporting of guidelines and thresholds

To better understand the implementation of guidelines in the care of HDFN-affected neonates, an additional analysis was performed to assess which publications specified guideline thresholds for exchange transfusions and simple RBC transfusions. Nine out of 18 (50%) studies reporting representative data on the frequency of exchange transfusions reported the guidelines and thresholds used. Among these, only 1 study reported on the method of exchange transfusions. The Turkish Neonatal Society guidelines to the approach, follow-up, and treatment of neonatal jaundice [67] was used in 2 studies, the American Academy of Pediatrics (AAP) guidelines [68] in 5 studies, and the remaining 2 studies provided details on the thresholds used without reference to a previously published guideline. Five out of 12 (42%) studies reporting representative data on the use of simple RBC transfusions reported the thresholds used. None of these studies provided a reference to a previously published guideline. None of the nonrepresentative studies reported the guidelines or thresholds for exchange transfusions (*n* = 9) or simple RBC transfusions (*n* = 8).

## Discussion

### Main findings

We found that the current clinical burden of HDFN, based on the use of exchange transfusions and simple RBC transfusions in neonates affected by Rh(D)- and/or K-mediated HDFN, is relatively high with a median exchange transfusion use of 27% in Rh(D)-mediated HDFN and 6% in K-mediated HDFN. We also found that the need for at least 1 simple RBC transfusion was required in 60% of Rh(D)-mediated HDFN and in 50% of K-mediated HDFN. In addition, a large variance in the rate of exchange transfusions and RBC transfusions in both Rh(D)-mediated HDFN and K-mediated HDFN was found, indicating that the rate of these procedures may vary widely between centers and countries and that a consensus on the management may be lacking. We were unable to determine the frequency of adverse events related to exchange transfusions and RBC transfusions. However, it was reported that delayed onset anemia occurred in 72% of infants with K-mediated HDFN, indicative that the effects of antenatal maternal alloimmunization can carry over into infancy and that clinicians should be aware of anti-K mediated inhibition of erythroid progenitors. We were unable to extract representative data on the occurrence of delayed onset anemia in Rh(D)-mediated HDFN, demonstrating that it may not be well recognized or reported in literature due to a false sense of security despite the persistence of maternal IgG in neonatal blood after birth. It should also be taken into consideration that most of the neonates with Rh(D)- and/or K-mediated HDFN in the included studies were born late preterm, thereby disrupting the intrauterine development. This was previously described elsewhere in more detail in the prenatal study from the same literature search [24].

### Strengths and limitations

There are several limitations to this study. Firstly, mostly studies from middle- to high-income countries and relatively large centers were included. No studies with a higher level of evidence (e.g., observational cohort studies or trials) from centers in low-income countries were available, which is a natural consequence of rare diseases and a general absence of centralization of prenatal care that is currently not feasible in many countries. More insight into performed treatments and clinical outcomes from centers with fewer cases and low-to-middle income countries is needed to more accurately determine the current postnatal treatment landscape and clinical outcomes of HDFN. Also the performed search and selection of studies may have been limited as the search was not performed on Scientific Electronic Library Online (Scielo) or African Index Medicus and only English language studies were selected. However, through including case series and case reports we were able to retrieve the currently available data from centers with fewer cases. Moreover, we were unable to identify the number of records found stratified per database and the number of duplicate records that were automatically removed. Additionally, we performed the search using ProQuest and were consequently unable to retrieve the specific search strategies for Medline, clinicaltrials.gov and EMBASE limiting the preferred transparency and repeatability of the study. Lastly, through selecting cases with Rh(D)-mediated HDFN or K-mediated HDFN, we were unable to determine the treatment landscape and clinical outcomes of other alloantibodies that may induce disease, such as anti-Rh(c) or anti-Rh(E).

Even though we employed minimal limits on study design, we were unable to accurately determine the frequency of alternative postnatal treatment options. Due to a lack of representative studies, a generally low methodological quality, and a limited number of cases in the included studies, we were unable to determine frequencies of neonatal IVIG, recombinant erythropoietin, or plasma exchange and plasmapheresis. Mostly case reports and case series reported on the use of these less frequently used treatment types, indicative of a potential low frequency. Also, we were unable to extract data on neonatal comorbidities (e.g., cardiac dysfunction, sepsis, necrotizing enterocolitis and bilirubin encephalopathy) or standard-of-care neonatal treatments (e.g., the need for (non-)mechanical ventilation or the use of umbilical venous catheters) that might have an effect on the clinical outcome of these sometimes severely ill patients. Thus, the evidence does reveal a significant lack of available information on both the employment of less frequently used treatments for HDFN, the use of other neonatal standard-of-care treatments, and the occurrence of comorbidities that may be due to premature delivery of HDFN-affected neonates. Also, using these inclusion and exclusion criteria, no studies on long-term neurodevelopmental outcome were included in this review.

Lastly, due to the descriptive nature of this rapid review and the consequent lack of interventions, we were unable to assess the level of heterogeneity between studies using the Chi^2^- and I^2^-statistic. However, a certain level of heterogeneity between studies may be expected, due to a large variety in included cases, transfusion rates and employed guidelines. Additionally, sociodemographic and geographical differences, that are commonly not reported on, may add to the level of heterogeneity.

### Implication of the findings

Great advances in the postnatal management of HDFN have been made since the very first exchange transfusion for neonatal icterus in 1924 [69]. As a consequence, the neonatal mortality rate among infants treated with exchange transfusions decreased from approximately 12% in the late 1940s and early 1950s [70, 71] to 0–6% in the twenty-first century [72–74]. In addition, the recently published 2022 AAP guidelines for the management of hyperbilirubinemia recommends to increase the total bilirubin threshold for exchange transfusions, potentially leading to another decrease in the rate of exchange transfusions and underlining the importance of intensive phototherapy [75]. Intensive phototherapy was found to be employed in nearly all of the reported cases in the included studies. The serious risk of complications associated with exchange transfusions, inherent to its invasive nature, should not be taken lightly. As previously stated, we were unable to extract data on adverse events associated with exchange transfusions in this literature search. But, in a recent retrospective study—a study that was excluded from this rapid review—of exchange transfusions performed over a 20-year period in the national referral center for HDFN in the Netherlands, thrombocytopenia (< 100 × 10^9^/L) occurred in > 90% of cases, of which 20% with a thrombocyte count < 25 × 10^9^/L, and leukopenia (< 5 × 10^9^/L) occurred in 71% of cases. Importantly, proven sepsis related to exchange transfusions occurred in 11% of neonates [73].

**Fig. 2:**
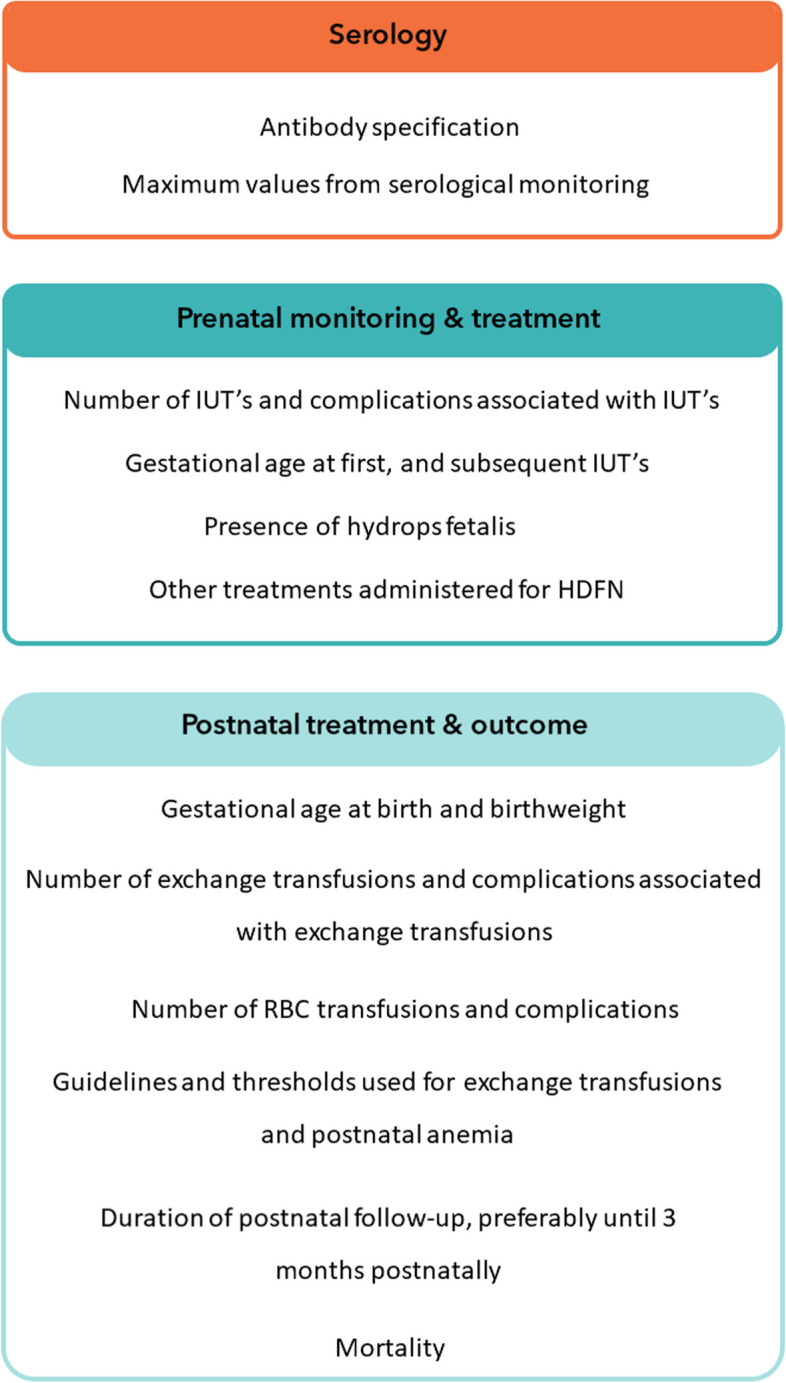
Recommended parameters for future studies. HDFN, hemolytic disease of the fetus and newborn; IUT, intrauterine transfusion; RBC, red blood cell

As previously mentioned, a large variance was found in the rate of exchange transfusions and simple RBC transfusions, suggestive of a lack of consensus on transfusions thresholds. Future studies ought to more accurately identify differences and similarities in the postnatal management strategies, as well as the available therapy options, and the clinical outcomes of those affected between centers in an international perspective. This information may be used to open a discussion, encourage international cooperation and, importantly, improve the care and outcomes of those affected. Lastly, we noted a shortage and inconsistency in the reporting of relevant data. For instance, approximately 50% of the studies did not report essential information on guidelines and thresholds used for exchange transfusions and simple RBC transfusions. Therefore, we have developed a list of parameters that are recommended to be reported in future studies, if available, as these parameters could nuance and provide a better framework for the studies’ findings (Fig. 2).

## Conclusion

In summary, we found that, although the neonatal mortality rate is nowadays low, the postnatal clinical burden of Rh(D)- and/or K-mediated HDFN remains relatively high, with a high need for exchange transfusions and simple RBC transfusions. Large differences between centers and countries may exist in the rate of exchange transfusions and simple RBC transfusions. We have identified several evidence gaps that should be addressed and provide an opportunity for future studies in an international perspective. Future studies should also report several vital parameters on guidelines and thresholds, methodology, and results to ensure an increase in the quality, validity, and replicability of the study.

### Supplementary Information

**Additional file 1: Appendix S1. **Search Strategy.** Appendix S2.** Inclusion and Exclusion Criteria.

**Additional file 2: Supplementary Table 1. **Research Questions. **Supplementary Table 2.** General Characteristics of Included Studies. **Supplementary Table 3.** Postnatal Treatments Reported in Studies With Representative Data. **Supplementary Table 4.** Delayed-onset Anemia, Hyperbilirubinemia, Neurodevelopmental Outcomes, and Adverse Events Reported in Studies With Representative Data. **Supplementary Table 5.** Overall Neonatal Mortality Associated With HDFN. **Supplementary Table 6.** Overall methodological quality score, defined per study.

**Additional file 3.**

## Data Availability

The datasets used and/or analysed during the current study available from the corresponding author on reasonable request.

## Abbreviations

HDFN: Hemolytic disease of the fetus and newborn
CHERRIES: Checklist for Reporting Results of Internet E-Surveys
IQR: Interquartile range
IUT: Intrauterine transfusion
IVIG: Intravenous immunoglobulins
JBI: Joanna Briggs Institute
MOOSE: Meta-analysis of Observational Studies in Epidemiology
PRISMA: Preferred Reporting Items for Systematic Reviews and Meta-Analyses
RCT: Randomized controlled trial
Rh: Rhesus
SD: Standard deviation
USA: United States of America
AAP: The American Academy of Pediatrics
