# Supplementary material for: Hemolytic disease of the fetus and newborn: rapid review of postnatal care and outcomes
Source: BMC Pregnancy Childbirth. 2023 Oct 18;23:738. doi: 10.1186/s12884-023-06061-y (PMC10583489; doi:10.1186/s12884-023-06061-y)
Supplement: Supplementary file 1 — Additional file 1: Appendix S1. Search Strategy. Appendix S2. Inclusion and Exclusion Criteria. [file 12884_2023_6061_MOESM1_ESM.pdf]

Appendix S1. Search Strategy

#	Description	Search string	Hits
S1	Disease state	(EMB.EXACT.EXPLODE("newborn hemolytic disease") OR MESH.EXACT.EXPLODE("Erythroblastosis, Fetal")) AND (TI,AB(HDFN OR ((hemolytic OR haemolytic) NEAR/3 (fetus OR newborn OR foetus)))) OR TI,AB("hemolytic disease of the fetus and newborn" OR "haemolytic disease of the fetus and newborn" OR "hemolytic disease of the foetus and newborn" OR "haemolytic disease of the foetus and newborn" OR "hemolytic disease of the newborn" OR "haemolytic disease of the newborn" OR HDFN) OR EMB.EXACT.EXPLODE("alloimmunization") OR ("maternal alloimmunization") OR ("red cell alloimmunization")	12,499[a]
S2	Intervention/treatment setting	EMB.EXACT("intrauterine blood transfusion") OR EMB.EXACT("fetal therapy") OR EMB.EXACT.EXPLODE("neonatal intensive care unit") OR MESH.EXACT.EXPLODE("Fetal Therapies") OR MESH.EXACT("Intensive Care Units, Neonatal") OR EMB.EXACT("immunoglobulin") OR MESH.EXACT("Immunoglobulins, Intravenous") OR EMB.EXACT.EXPLODE("plasma exchange") OR MESH.EXACT.EXPLODE("Plasma Exchange") OR ("therapeutic plasma exchange") OR ("neonatal transfusion") OR EMB.EXACT.EXPLODE("exchange blood transfusion") OR ("neonatal exchange transfusion")	23,3001[a]
S3	Clinical outcomes	MESH.EXACT("Mortality") OR EMB.EXACT("mortality") OR EMB.EXACT("infant mortality") OR MESH.EXACT.EXPLODE("Infant Mortality") OR MESH.EXACT(Morbidity) OR EMB.EXACT(morbidity) OR MESH.EXACT.EXPLODE("Anemia, Neonatal") OR MESH.EXACT.EXPLODE("Hyperbilirubinemia") OR MESH.EXACT("Fetal Blood") OR MESH.EXACT("Perinatal Death") OR MESH.EXACT("Fetal Death") OR EMB.EXACT("newborn jaundice") OR EMB.EXACT("neonatal hyperbilirubinemia") OR EMB.EXACT("anemia") OR EMB.EXACT("hyperbilirubinemia") OR EMB.EXACT.EXPLODE("perinatal morbidity") OR EMB.EXACT("newborn death") OR EMB.EXACT("fetus death") OR EMB.EXACT("rhesus incompatibility") OR (late NEAR/3	6,724,924[a]

		anemia) OR TI,AB(complication*) OR TI,AB(adverse /NEAR1 (effect* OR reaction* OR event*)) OR MESH.EXACT("Hydrops Fetalis") OR EMB.EXACT.EXPLODE("edema") OR EMB.EXACT.EXPLODE("fetus hydrops") OR MESH.EXACT.EXPLODE("Premature Birth") OR EMB.EXACT.EXPLODE("prematurity") OR (emergency NEAR/2 childbirth OR delivery OR cesarean section) OR MESH.EXACT.EXPLODE("Respiration, Artificial") OR EMB.EXACT("artificial ventilation") OR MESH.EXACT("Heart Failure") OR EMB.EXACT("heart failure")	
S4	Combined disease State and outcomes of interest	S1 AND S2	1903[b]
S5		S1 AND S3	3619[b]
S6	Total hits	S4 OR S5	5069[a]
S7	Final hits published between January 1, 2005, and March 10, 2021	S6 AND publication date (>2004)	2518[b]

[a]Duplicates were removed from the search but included in the result count.

[b]Duplicates were removed from the search and from the result count.

Appendix S2. Inclusion and Exclusion Criteria[a]

	Inclusion criteria	Exclusion criteria
Population	• Infants or children experiencing or having experienced HDFN caused by Rh incompatibility<br>• Antigen status: Rh(D) or Kell antigen	• Infants and children who did not experience HDFN and newborns with ABO incompatibility<br>• Antigen status: c, e, E, Duffy (Fy), Kidd (Jk), MNS (S), or Gerbich antigen
Intervention	• Any or none	• Not applicable
Comparator	• Any or none	• Not applicable
Outcomes	• Treatment patterns<br>• Clinical outcomes<br>• Treatment efficacy	• Not applicable
Study design	• Observational studies (retrospective or prospective), including cohort, case-control, or cross-sectional studies<br>• Trials (randomized or nonrandomized)<br>• Modeling studies<br>• Systematic reviews of cohort studies (for identification of primary studies only)<br>• Case reports and case series	• Notes, editorials, or commentaries<br>• Nonsystematic reviews
Other	• Journal articles<br>• Human subjects<br>• English language<br>• Studies published between January 1, 2005, and March 10, 2021	• Indexed conference abstracts<br>• Publication types not of interest<br>• Animal or preclinical studies<br>• Non-English language<br>• Studies published before January 1, 2005

HDFN, hemolytic disease of the fetus and newborn; PICOS, Population, Intervention, Comparison, Outcomes, and Study; Rh, Rhesus.

[a]PICOS design was used as a framework to formulate eligibility criteria.
