# Supplementary material for: Hemolytic disease of the fetus and newborn: rapid review of postnatal care and outcomes
Source: BMC Pregnancy Childbirth. 2023 Oct 18;23:738. doi: 10.1186/s12884-023-06061-y (PMC10583489; doi:10.1186/s12884-023-06061-y)
Supplement: Supplementary file 2 — Additional file 2: Supplementary Table 1. Research Questions. Supplementary Table 2. General Characteristics of Included Studies. Supplementary Table 3. Postnatal Treatments Reported in Studies With Representative Data. Supplementary Table 4. Delayed-onset Anemia, Hyperbilirubinemia, Neurodevelopmental Outcomes, and Adverse Events Reported in Studies With Representative Data. Supplementary Table 5. Overall Neonatal Mortality Associated With HDFN. Supplementary Table 6. Overall methodological quality score, defined per study. [file 12884_2023_6061_MOESM2_ESM.docx]

**Supplementary Table 1. Research Questions**

| **PICO categories** | **Research question 1** | **Research question 2** |
| --- | --- | --- |
| **P**atient | In neonates | In neonates |
| **I**ntervention | Affected by Rh(D)-mediated and/or K-mediated HDFN | Affected by Rh(D)-mediated and/or K-mediated HDFN |
| **C**omparison | Not applicable | Not applicable |
| **O**utcome | What is the: Mortality rate, gestational age at birth, complication rate and rate of comorbidities (cardiac dysfunction, respiratory distress syndrome, necrotizing enterocolitis, sepsis, bilirubin encephalopathy) | What is the: Rate of exchange transfusions, phototherapy, RBC transfusions, standard-of-care neonatal treatments ((non-)mechanical ventilation and use of umbilical venous catheters) |

HDFN, hemolytic disease of the fetus and newborn. RBC, red blood cell

**Supplementary Table 2. General Characteristics of Included Studies**

| **Study** | **Study type** | **Data source** | **Sample size** | **Groups** | **Group size** | **Data collection period** | **Country** | **Funding source** |
| --- | --- | --- | --- | --- | --- | --- | --- | --- |
| Akdag 2012 (27) | Case report | Medical record | 1 | Neonate, K-mediated HDFN | 1 | NR | Turkey | Not funded |
| Azavkli 2020 (61) | Retrospective cohort | Medical records | 110 | Neonate, Rh(D)-mediated HDFN | 42 | January 2015-July 2018 | Turkey | Not reported |
| Bek 2019 (28) | Case report | Medical record | 1 | Neonate, Rh(D)-mediated HDFN | 1 | NR | Turkey | Not reported |
| Bennardello 2013 (29) | Questionnaire | Questionnaire | 1661 | Neonates, Rh(D)-mediated HDFN | 111 | NR | Italy | Not reported |
| Bi 2019 (30) | Prospective cohort | Prospective registry | 18 | Rh(D)-mediated HDFN | 5 | January 2017-June 2019 | People’s Republic of China | Not reported |
| Brumbaugh 2011 (31) | Case report | Medical record | 1 | Neonate, K-mediated HDFN | 1 | NR | USA | Not funded |
| Chatziantoniou 2017 (32) | Retrospective cohort | Medical records | 130 | Neonates, Rh(D)-mediated HDFN | 22 | June 2006-June 2013 | UK | Not reported |
|  |  |  |  | Neonate, K-mediated HDFN | 1 |  |  |  |
| Colpo 2017 (33) | Case report | Medical record | 1 | Neonate, Rh(D)-mediated HDFN | 1 | NR | Italy | Not funded |
| De Assuncão 2016 (34) | Prospective cohort | Prospective registry | 13 | Neonates, Rh(D)-mediated HDFN | 13 | NR | Brazil | Not reported |
| Gottvall 2008 (35) | Retrospective cohort | Medical records | 78,145 | Neonates, Rh(D)-mediated HDFN | 71 | January 1992-December 2005 | Sweden | Not reported |
| Gudlaugsson 2020 (36) | Retrospective cohort | Medical records | 132 | Neonates, Rh(D)-mediated HDFN | 85 | 1996-2015 | Iceland | Not reported |
| Haider 2020 (37) | Case report | Medical record | 1 | Neonate, Rh(D)-mediated HDFN | 1 | NA | Pakistan | Not funded |
| Harper 2006 (38) | Prospective cohort | Prospective registry | 18 | Neonates, Rh(D)-mediated HDFN | 3 | July 1985-October 1995 | USA | Supported by the General Clinical Research Centers Program,  grant NCRR RR00059; the National Institutes of Health, grant P01  HL46925; and the Children’s Miracle Network Telethon of Iowa. |
|  |  |  |  | Neonates, K-mediated HDFN | 2 |  |  |  |
| Hassan 2019 (39) | Case report | Medical record | 1 | Neonate, Rh(D)-mediated HDFN | 1 | NA | Malaysia | Not funded |
| Karagol 2012 (40) | Retrospective cohort | Medical records | 106 | Neonates, K-mediated HDFN | 5 | January 2005-December 2010 | Turkey | Not reported |
| Kriplani 2007 (41) | Case series | Medical records | 4 | Neonates, Rh(D)-mediated HDFN | 4 | NR | India | Not reported |
| Lakhwani 2011 (42) | Case report | Medical record | 1 | Neonate, K-mediated HDFN | 1 | NR | Spain | Not reported |
| Levy-Zauberman 2011 (43) | Case report | Medical record | 1 | Neonate, Rh(D)-mediated HDFN | 1 | NR | France | Not funded |
| Lieberman 2020 (44) | Retrospective cohort | Medical records | 128 | Neonates, Rh(D)-mediated HDFN | 18 | November 2020-June 2017 | Canada | Laboratory Medicine and Pathology  Summer Student Research Grant,  University of Toronto; Canadian Blood  Services, Transfusion Medicine Research  Program Support Award |
|  |  |  |  | Neonates, K-mediated HDFN | 2 |  |  |  |
| Manoura 2007 (45) | Case report | Medical record | 1 | Neonate, K-mediated HDFN | 1 | NR | Greece | Not reported |
| Matijevic 2005 (46) | Retrospective cohort | Medical records | 23 | Neonates, Rh(D)-mediated HDFN | 15 | January 1997-January 2003 |  | Not reported |
|  |  |  |  | Neonate, K-mediated HDFN | 1 |  |  |  |
| Mayer 2018 (47) | Case series | Medical records | 3 | Neonate, Rh(D)-mediated HDFN | 1 | NR | Germany | Not reported |
|  |  |  |  | Neonate, K-mediated HDFN | 1 |  |  |  |
| Meraj 2015 (48) | Retrospective cohort | Medical records | 8 | Neonates, Rh(D)-mediated HDFN | 5 | January 2001-December 2001 | Pakistan | Not reported |
| Navarro 2009 (49) | Case series | Medical records | 3 | Neonate, Rh(D)-mediated HDFN | 1 | NR | USA | Not reported |
| Nwogu 2018 (50) | Case series | Medical records | 5 | Neonates, Rh(D)-mediated HDFN | 3 | November 2011-December 2015 | USA | Not reported |
|  |  |  |  | Neonate, K-mediated HDFN | 1 |  |  |  |
|  |  |  |  | Neonate, Rh(D)- and K-mediated HDFN | 1 |  |  |  |
| Palfi 2006 (51) | Case report | Medical record | 1 | Neonate, Rh(D)-mediated HDFN | 1 | NA | Sweden | Not reported |
| Phung 2018 (52) | Retrospective cohort | Medical records | 106 | Neonates, isolated Rh(D)-mediated HDFN | 27 | 1999-2015 | France | Not reported |
| Raguz 2020 (26) | Retrospective | Medical records | 29,663 pregnancies | Infants, Rh(D)-mediated HDFN | 59 | 2000-2019 | Bosnia and Herzegovina | Not reported |
|  |  |  |  | Infants, K-mediated HDFN | 5 |  |  |  |
| Rahimi-Sharbaf 2007 (53) | Case report | Medical record | 1 | Neonate, Rh(D)-mediated HDFN | 1 | NA | Iran | Not reported |
| Rath 2011 (54) | Retrospective | Medical records | 191 | (Near)-term neonates, Rh(D)-mediated HDFN | 157 | January 2000-December 2008 | The Netherlands | Not reported |
|  |  |  |  | (Near)-term neonates, K-mediated HDFN | 34 |  |  |  |
| Rath 2013 (55) | Retrospective cohort | Medical records | 125 | Neonates, Rh(D)-mediated HDFN | 103 | January 2000-October 2011 | The Netherlands | Not reported |
| Ree 2019 (56) | Retrospective cohort | Medical records | 298 | Infants, Rh(D)-mediated HDFN | 224 | January 2006-Jaunary 2018 | The Netherlands | Not reported |
|  |  |  |  | Infants, K-mediated HDFN | 39 |  |  |  |
| Ree 2020a (20) | Retrospective cohort | Medical records | 317 | Infants, Rh(D)-mediated HDFN | 2 | January 2000-December 2016 | The Netherlands | Not reported |
|  |  |  |  | Infant, K-mediated HDFN | 1 |  |  |  |
| Ree 2020b (57) | Retrospective cohort | Medical records | 235 | Neonates, Rh(D)-mediated HDFN | 189 | January 2005-December 2018 | The Netherlands | Not reported |
|  |  |  |  | Neonates, K-mediated HDFN | 46 |  |  |  |
| Ruma 2007 (58) | Multicenter case series | Medical records | 9 | Infants, Rh(D)-mediated HDFN | 5 | 1996-2005 | USA | Not reported |
|  |  |  |  | Infants, K-mediated HDFN | 4 |  |  |  |
| Sainio 2015 (59) | Retrospective cohort | Finnish Red Cross Blood Service Database and questionnaires | 104 | Neonates, Rh(D)-mediated HDFN | 86 | 2003-2012 | Finland | Not funded |
|  |  |  |  | Neonates, K-mediated HDFN | 6 |  |  |  |
| Santos 2013 (60) | RCT | Prospective registry | 92 | Infants, Rh(D)-mediated HDFN | 92 | April 2006-June 2009 | Brazil | Not reported |
| Simonazzi 2016 (62) | Case series | Medical records | 4 | Neonates, Rh(D)-mediated HDFN | 3 | NR | Italy | Not reported |
| Takci 2013 (16) | Retrospective cohort | Medical records | 30 | Hydropic infants, Rh(D)-mediated HDFN | 30 | January 2001-June 2012 | Turkey | Not reported |
| Temel Yuksel 2019 (63) | Prospective cohort | Prospective registry | 17 | Neonates, Rh(D)-mediated HDFN | 17 | January 2018-June 2019 | Turkey | Not reported |
| Tiblad 2011 (64) | Retrospective cohort | Medical records, local databases and the Swedish Quality Register on Neonatal Intensive Care | 84 | Neonates, Rh(D)-mediated HDFN | 67 | June 1990-June 2010 | Sweden | Not reported |
|  |  |  |  | Neonates, K-mediated HDFN | 9 |  |  |  |
| Walsh 2013 (65) | Retrospective cohort | Medical records | 102 | Neonates, Rh(D)-mediated HDFN | 26 | January 1, 1996-December 31, 2011 | Republic of Ireland | Not funded |
|  |  |  |  | Neonates, K-mediated HDFN | 11 |  |  |  |
| Xu 2013 (66) | Case report | Medical record | 1 | Neonate | 1 | NA | People’s Republic of China | Not reported |

HDFN, hemolytic disease of the fetus and newborn; NA, not applicable; NR, not reported; RCT, randomized controlled trial; Rh(D), Rhesus factor D.

**Supplementary Table 3. Postnatal Treatments Reported in Studies With Representative Data**

| **Citation** | **Study design** | **Population** | **Rh(D) or K** | **Treatment type** | **Treatment (%)** |
| --- | --- | --- | --- | --- | --- |
| **Phototherapy** | | | | | |
| Azavkli 2020 (61) | Retrospective cohort | 42 | Rh(D) | IUT, phototherapy, RBC transfusion | 100 |
| Gottvall 2008 (35) | Retrospective cohort | 71 | Rh(D) | Maternal plasma exchange and/or high-dose IVIG, neonatal exchange transfusion, phototherapy | 15.5 |
| Karagol 2012 (40) | Retrospective cohort | 5 | K | IVIG, neonatal exchange transfusion, phototherapy | 80 |
| Kriplani 2007 (41) | Case series | 4 | Rh(D) | Maternal IVIG, IUT, phototherapy | 50 |
| Lieberman 2020 (44) | Retrospective cohort | 18 | Rh(D) | Neonatal IVIG, neonatal exchange transfusion, phototherapy, RBC transfusion | 67 |
|  |  | 2 | K | Neonatal IVIG, phototherapy, RBC transfusion | 100 |
| Matijevic 2005 (46) | Retrospective cohort | 16 | Rh(D) & K | IUT, neonatal exchange transfusion, phototherapy | 75 |
| Meraj 2015 (48) | Retrospective cohort | 5 | Rh(D) | Exchange transfusion, phototherapy, RBC transfusion | 100 |
| Phung 2018 (52) | Retrospective cohort | 27 | Rh(D) | IUT, neonatal exchange transfusion, phototherapy, RBC transfusion, delayed cord clamping | 78.6 |
| Raguz 2020 (26) | Retrospective cohort | 59 | Rh(D) | Exchange transfusion, phototherapy | 100 |
|  |  | 5 | K | Exchange transfusion, phototherapy | 100 |
| Rath 2011 (54) | Retrospective cohort | 157 | Rh(D) | IUT, neonatal exchange transfusion, RBC transfusion, phototherapy | 98 |
|  |  | 34 | K | IUT, neonatal exchange transfusion, RBC transfusion, phototherapy | 91 |
| Rath 2013 (55)b | Retrospective cohort | 103 | Rh(D) | Maternal IVIG, IUT, neonatal exchange transfusion, phototherapy, RBC transfusion | 98 |
| Ree 2020b (57) | Retrospective cohort | 189 | Rh(D) | Maternal IVIG, IUT, neonatal exchange transfusion, phototherapy, RBC transfusion | 100 |
|  |  | 46 | K | Maternal IVIG, IUT, phototherapy, RBC transfusion | 100 |
| Ruma 2007 (58) | Case series | 9 | Rh(D) & K | IVIG, IUT, phototherapy, RBC transfusion | 22 |
| Santos 2013 (60) | RCT | 46 | Rh(D) | IUT, neonatal IVIG, neonatal exchange transfusion, phototherapy | 100 |
|  |  | 46 | Rh(D) | IUT, neonatal exchange transfusion, phototherapy | 100 |
| Temel Yuksel 2019 (63) | Prospective cohort | 17 | Rh(D) | IUT, phototherapy | 100 |
| **Exchange transfusion** | | | | | |
| Azavkli 2020 (61) | Retrospective cohort | 42 | Rh(D) | IUT, phototherapy, RBC transfusion | 58.8 |
| Bennardello 2013 (29) | Questionnaire | 111 | Rh(D) | Maternal IVIG, IUT | 28.8 |
| Bi 2019 (30) | Prospective cohort | 5 | Rh(D) | Neonatal exchange transfusion only | 100 |
| Gottvall 2008 (35) | Retrospective cohort | 71 | Rh(D) | Maternal plasma exchange and/or high-dose IVIG, neonatal exchange transfusion, phototherapy | 19.6 |
| Gudlaugson 2020 | Retrospective cohort | 85 | Rh(D) | Exchange transfusion only | 100 |
| Karagol 2012 (40) | Retrospective cohort | 5 | K | IVIG, exchange transfusion, phototherapy | 80 |
| Lieberman 2020 (44) | Retrospective cohort | 18 | Rh(D) | Neonatal IVIG, neonatal exchange transfusion, phototherapy, RBC transfusion | 6 |
|  |  | 2 | K | Neonatal IVIG, phototherapy, RBC transfusion | 0 |
| Matijevic 2005 (46) | Retrospective cohort | 16 | Rh(D) & Kell | IUT, exchange transfusion, phototherapy | 62.5 |
| Meraj 2015 (48) | Retrospective cohort | 5 | Rh(D) | Exchange transfusion, phototherapy, RBC transfusion | 20 |
| Phung 2018 (52) | Retrospective cohort | 27 | Rh(D) | IUT, neonatal exchange transfusion, phototherapy, RBC transfusion, delayed cord clamping | 42.9 |
| Raguz 2020 (26) | Retrospective cohort | 59 | Rh(D) | Exchange transfusion, phototherapy | 26 |
|  |  | 5 | K | Exchange transfusion, phototherapy | 20 |
| Rath 2011 (54) | Retrospective cohort | 157 | Rh(D) | IUT, neonatal exchange transfusion, RBC transfusion, phototherapy | 6 |
|  |  | 34 | K | IUT, neonatal exchange transfusion, RBC transfusion, phototherapy | 62 |
| Rath 2013 (55)b | Retrospective cohort | 103 | Rh(D) | Maternal IVIG, IUT, neonatal exchange transfusion, phototherapy, RBC transfusion | 53.4 |
| Ree 2019 (56) | Retrospective cohort | 148 | Rh(D) | IUT, RBC transfusion | 20 |
|  |  | 76 | Rh(D) | RBC transfusion | 18 |
| Ree 2020b (57) | Retrospective cohort | 189 | Rh(D) | Maternal IVIG, IUT, neonatal exchange transfusion, phototherapy, RBC transfusion | 26.5 |
|  |  | 46 | K | Maternal IVIG, IUT, phototherapy | 0 |
| Santos 2013 (60) | RCT | 46 | Rh(D) | IUT, neonatal IVIG, neonatal exchange transfusion, phototherapy | 13 |
|  |  | 46 | Rh(D) | IUT, neonatal exchange transfusion, phototherapy | 15.2 |
| Takci 2013 (16) | Retrospective cohort | 30 | Rh(D) and K | IUT, neonatal exchange transfusion, RBC transfusion, parenteral nutrition, oral ursodeoxycholic acid (phenobarbital), oral erythromycin | 90 |
| Temel Yuksel 2019 (63) | Prospective cohort | 17 | Rh(D) | IUT, phototherapy | 41.1 |
| **RBC transfusion** | | | | | |
| Azavkli 2020 (61) | Retrospective cohort | 42 | Rh(D) | IUT, phototherapy, RBC transfusion | 73.5 |
| Lieberman 2020 (44) | Retrospective cohort | 18 | Rh(D) | Neonatal IVIG, exchange transfusion, phototherapy, RBC transfusion | 17 |
|  |  | 2 | K | Neonatal IVIG, phototherapy, RBC transfusion | 50 |
| Meraj 2015 (48) | Retrospective cohort | 5 | Rh(D) | Exchange transfusion, phototherapy, RBC transfusion | 60 |
| Phung 2018 (52) | Retrospective cohort | 27 | Rh(D) | IUT, neonatal exchange transfusion, phototherapy, RBC transfusion, delayed cord clamping | 67.9 |
| Rath 2011 (54) | Retrospective cohort | 157 | Rh(D) | IUT, neonatal exchange transfusion, RBC transfusion, phototherapy | 62 |
|  |  | 34 | K | IUT, neonatal exchange transfusion, RBC transfusion, phototherapy | 72 |
| Rath 2013 (55)b | Retrospective cohort | 103 | Rh(D) | Maternal IVIG, IUT, neonatal exchange transfusion, phototherapy, RBC transfusion | 78 |
| Ree 2019 (56) | Retrospective cohort | 148 | Rh(D) | IUT, RBC transfusion | 20 |
|  |  | 76 | Rh(D) | RBC transfusion | 18 |
| Ree 2020b (57) | Retrospective cohort | 189 | Rh(D) | Maternal IVIG, IUT, neonatal exchange transfusion, phototherapy, RBC transfusion | 26.5 |
|  |  | 46 | K | Maternal IVIG, IUT, phototherapy, RBC transfusion | 71.7 |
| Ruma 2007 (58) | Case series | 9 | Rh(D) & K | IVIG + IUT, phototherapy, RBC transfusion | 56 |
| Takci 2013 (16) | Retrospective cohort | 30 | Rh(D) & K | IUT, neonatal exchange transfusion, RBC transfusion, parenteral nutrition, oral ursodeoxycholic acid (phenobarbital), oral erythromycin | 73.3 |
| **Neonatal IVIG** | | | | | |
| Lieberman 2020 (44) | Retrospective cohort | 18 | Rh(D) | Neonatal IVIG, exchange transfusion, phototherapy, RBC transfusion | 28 |
|  |  | 2 | K | Neonatal IVIG, phototherapy, RBC transfusion | 50 |
|  |  | 46 | Rh(D) | IUT, neonatal exchange transfusion, phototherapy | 0 |
| **Other treatments** | | | | | |
| Brumbaugh 2011 (31) | Case report | 1 | K | Neonatal IVIG, platelet, plasma, and cryoprecipitate transfusion, chelation therapy | 100 |
| Chatziantoniou 2017 (32) | Retrospective cohort | 22 | Rh(D) | IUT + exchange transfusion + phototherapy + IVIG | 4.5 |
|  |  |  |  | Exchange transfusion + phototherapy + IVIG | 13.6 |
|  |  |  |  | Phototherapy + IVIG | 31.8 |
| Karagol 2012 (40) | Retrospective cohort | 5 | K | IVIG, exchange transfusion, phototherapy | 20 |
| Levy-Zauberman 2011 (43) | Case report | 1 | Rh(D) | IUT, phototherapy, platelets, frozen plasma transfusion | 100 |
| Manoura 2007 (45) | Case report | 1 | K | Recombinant erythropoietin and oral iron supplement | 100 |
| Takci 2013 (16) | Retrospective cohort | 30 | Rh(D) & K | IUT, neonatal exchange transfusion, RBC transfusion, parenteral nutrition, oral ursodeoxycholic acid (phenobarbital), oral erythromycin | 40 |

HDFN, hemolytic disease of the fetus and newborn; IQR, interquartile range; IUT, intrauterine transfusion; IVIG, intravenous immunoglobulin; RBC, red blood cell; RCT, randomized controlled trial; Rh(D), Rhesus factor D; SD, standard deviation.

**Supplementary Table 4. Delayed-onset Anemia, Hyperbilirubinemia, Neurodevelopmental Outcomes, and Adverse Events Reported in Studies With Representative Data**

| **Citation** | | **Study design** | | **Patient group** | | **N** | **Treatment (%)** | | **Events (%)** | |
| --- | --- | --- | --- | --- | --- | --- | --- | --- | --- | --- |
| **Delayed onset anemia** | | | | | | | | | | |
| Ree 2019 (56) | | Retrospective cohort | | Infants, K-mediated HDFN | | 39 | IUT (90), exchange transfusion (72) | | 72 | |
| **Hyperbilirubinemia** | | | | | | | | | | |
| Meraj 2015 (48) | Retrospective cohort | | Neonates, Rh(D)-mediated HDFN | | 5 | | | Neonatal exchange transfusion (20), phototherapy (100),  RBC transfusion (60) | 100 |  |
| **Adverse events** | | | | | | | | | |  |
| Bi 2019 (30) | Prospective cohort | | Neonates, Rh(D)-mediated HDFN | | 5 | | | Exchange therapy (100) | No adverse events reported |  |
| de Assunção 2016 (33) | Prospective cohort | | Neonates, Rh(D)-mediated HDFN | | 13 | | | IUT (100) | Kernicterus (7.7)  Pulmonary hypertension (7.7)  RDS (7.7)  BPD (7.7)  Sepsis (15.4) |  |
| Takci 2013 (16) | Retrospective cohort | | Neonates, Rh(D)-mediated HDFN | | 30 | | | IUT (76.7), exchange therapy (90), RBC transfusion (73.3), parenteral nutrition (40) | Sepsis (13.3) |  |
| Temel Yuksel 2019 (63) | Prospective cohort | | Neonates, Rh(D)-mediated HDFN | | 17 | | | IUT (100), exchange therapy (41.1), phototherapy (100) | Respiratory distress (17.6)  Cord pH <7.2 (11.7)  NICU admission (70.5) |  |
| **Neurodevelopmental outcomes** | | | | | | | | | |  |
|  | Gestational age at first IUT (weeks) | | Hemoglobin at first IUT (g/dL) | | Total no. IUTs | | | Age at neurodevelopmental assessment (years) | Result of neurodevelopmental assessment |  |
| Harper 2006 (38) | | | | | | | | | |  |
| Case #3: Rh(D) | 22.0 | | 3.3 | | 5 | | | 12.4 | Static encephalopathy and cerebral palsy. Bilateral lower extremity paresis and atrophy, dystonic posturing of the right hand, and monocular esotropia |  |
| Case #7: K | 22.0 | | 1.8 | | 7 | | | 9.5 | No reported abnormalities |  |
| Case #10: Rh(D) | 23.5 | | 2.7 | | 6 | | | 6.2 | No reported abnormalities |  |
| Case #13: K | 23.5 | | 2.7 | | 6 | | | 6.2 | No reported abnormalities |  |
| Case #15: Rh(D) | 23.4 | | 4.0 | | 5 | | | 4.7 | No reported abnormalities |  |
| Levy-Zauberman 2011 (43) | | | | | | | | | |  |
| Rh(D) | 21.7 | | 2.5 | | 4 | | | 1.5 | No clinically detectable motor or cognitive impairment |  |
| Simonazzi 2016 (62) | | | | | | | | | |  |
| Case 2: Rh(D) | 23 | | 1.6 | | 6 | | | 12.0 | No reported abnormalities |  |
| Case 3: Rh(D) | 16 (intraperitoneal)  21 (intravascular) | | 2.6 | | 4 intraperitoneal  5 intravascular | | | 2.0 | Neurodevelopment delay, in particular truncal ataxia |  |

BPD, bronchopulmonary dysplasia; HDFN, hemolytic disease of the fetus and newborn; IUT, intrauterine transfusion; IVIG, intravenous immunoglobulin; NEC, necrotizing enterocolitis; NICU, neonatal intensive care unit; RBC, red blood cell; RDS, respiratory distress syndrome; Rh(D), Rhesus factor D.

**Supplementary Table 5. Overall Neonatal Mortality Associated With HDFN**

| **Citation** | **Study design** | **Patient group** | **N** | **Treatment (%)** | **Mortality rate (%)** |
| --- | --- | --- | --- | --- | --- |
| Azavkli 2020 (61) | Retrospective cohort | Neonates, Rh(D)-mediated HDFN | 42 | IUT (100), phototherapy (100), exchange transfusion (58.8) | 4.8 |
| Chatziantoniou 2017 (32) | Retrospective cohort | Neonate, Rh(D)-mediated HDFN | 1 | No treatment | 0 |
|  |  | Neonates, Rh(D)-mediated HDFN | 22 | IUT + exchange transfusion + phototherapy + IVIG (4.5)  Exchange transfusion + phototherapy + IVIG (3.6)  Phototherapy + IVIG (31.8) | 0 |
| Gotvall 2008 (35) | Retrospective cohort | Neonates, Rh(D)-mediated HDFN | 71 | Exchange transfusion (29.5), phototherapy (15.5) | 0 |
| Gudlaugsson 2020 (36) | Retrospective cohort | Neonates, Rh(D)-mediated HDFN | 135 | Exchange transfusion (96), no treatment (4) | 2.2 |
| Lieberman 2020 (44) | Retrospective cohort | Neonates, Rh(D)-mediated HDFN | 18 | Neonatal IVIG (28), neonatal exchange transfusion (6), phototherapy (67), RBC transfusion (17) | 0 |
|  |  | Neonates, K-mediated HDFN | 2 | Neonatal IVIG (50), phototherapy (50), RBC transfusion (50) | 0 |
| Ree 2020a (20) | Retrospective cohort | Neonate, Rh(D)-mediated HDFN | 1 | IUT, neonatal exchange transfusion, RBC transfusion | 0 |
|  |  | Neonate, Rh(D)-mediated HDFN | 1 | IUT, RBC transfusion | 0 |
|  |  | Neonate, K-mediated HDFN | 1 | IUT, neonatal IVIG | 100 |
| Sainio 2015 (59) | Retrospective cohort | Neonates, Rh(D)-mediated HDFN | 86 | IUT (100) | 1.2 |
| Takci 2013 (16) | Retrospective cohort | Hydropic infants, Rh(D)-mediated HDFN | 30 | IUT (76.7), neonatal exchange transfusion (90%), RBC transfusion (73.3), parenteral nutrition (40), oral ursodeoxycholic acid (± phenobarbital) (10), oral erythromycin (3.3) | 50 |
| Temel Yuksel 2019 (63) | Prospective cohort | Neonates, Rh(D)-mediated HDFN | 17 | IUT (100), neonatal exchange transfusion (41.1), phototherapy (100) | 5.8 |
| Walsh 2013 (65) | Retrospective cohort | Neonates, K-mediated HDFN | 11 | IUT (100) | 0 |
| Hassan 2019 (39) | Case report | Neonate, Rh(D)-mediated HDFN | 1 | Neonatal IVIG, RBC transfusion | 100 |

HDFN, hemolytic disease of the fetus and newborn; IUT, intrauterine transfusion; IVIG, intravenous immunoglobulin; RBC, red blood cell; Rh(D), Rhesus factor D

**Supplementary Table 6. Overall methodological quality score, defined per study**

| **Citation** | **Overall methodological quality score** |
| --- | --- |
| **Case reports assessed with JBI Critical Appraisal Checklist for Case Reports** | |
| Akdag 2012 (27) | **7/8** |
| Bek 2019 (28) | **7/8** |
| Brumbaugh 2011 (31) | **8/8** |
| Colpo 2017 (33) | **8/8** |
| Haider 2020 (37) | **6/8** |
| Hassan 2019 (39) | **5/8** |
| Lakhwani 2011 (42) | **8/8** |
| Levy-Zauberman 2011 (43) | **8/8** |
| Manoura 2007 (45) | **3/8** |
| Palfi 2006 (51) | **8/8** |
| Rahimi-Sharbaf 2007 (53) | **4/8** |
| Xu 2013 (66) | **8/8** |
| **Case series assessed with JBI Critical Appraisal Checklist for Case Series** | |
| Kriplani 2007 (41) | **5/9** |
| Mayer 2018 (47) | **4/9** |
| Navarro 2009 (49) | **5/9** |
| Nwogu 2018 (50) | **8/9** |
| Ruma 2007 (58) | **10/10** |
| Simonazzi 2016 (62) | **5/9** |
| **Retrospective Cohort Studies assessed using the Newcastle-Ottawa Scale** | |
| Åžavkli 2020 (61) | **Good (8)** |
| Chatziantoniou 2017 (32) | **Good (8)** |
| Gottvall 2008 (35) | **Good (8)** |
| Gudlaugsson 2020 (36) | **Good (8)** |
| Karagol 2012 (40) | **Good (8)** |
| Lieberman 2020 (44) | **Good (8)** |
| Matijevic 2005 (46) | **Fair (6)** |
| Meraj 2015 (48) | **Fair (6)** |
| Phung 2018 (52) | **Fair (7)** |
| Raguz 2020 (26) | **Fair (7)** |
| Rath 2013 (55)b | **Good (8)** |
| Ree 2019 (56) | **Good (8)** |
| Ree 2020a (20) | **Good (8)** |
| Ree 2020b (57) | **Good (8)** |
| Sainio 2015 (59) | **Good (8)** |
| Takci 2013 (16) | **Fair (7)** |
| Tiblad 2011 (64) | **Good (8)** |
| Walsh 2013 (65) | **Good (8)** |
| Rath 2011 (54) | **Good (8)** |
| **Prospective Cohort Studies assessed using the Newcastle-Ottawa Scale** | |
| Bi 2019 (30) | **Good (7)** |
| de Assunção 2016 (33) | **Good (8)** |
| Temel 2019 | **Good (8)** |
| Harper 2006 (38) | **Good (8)** |
| **Questionnaire study assessed using the CHERRIES-checklist** | |
| Bennardello 2013 (29) | **Good** |

**References of the 43 studies included in the systematic review**

16. Takcı S, Alarcon-Martinez T, Bozkaya D, Yiğit Ş, Korkmaz A, Yurdakök M. Cholestasis in infants with immune hydrops fetalis. Turk J Pediatr. 2013;55(6):616-9.

20. Ree IMC, de Grauw AM, Bekker V, de Haas M, Te Pas AB, Oepkes D, et al. Necrotizing enterocolitis in haemolytic disease of the newborn: a retrospective cohort study. Vox Sang. 2020;115(2):196-201.

26. Raguz MJ, Prce Z, Bjelanovic V, Bjelanovic I, Dzida S, Mabic M. 20 Years of Follow-up Alloimmunization and Hemolytic Disease in Newborn: Has Anything Changed in the Field Over the Years? Klin Padiatr. 2020;232(6):314-20.

27. Akdağ A, Erdeve O, Uraş N, Simşek Y, Dilmen U. Hydrops Fetalis due to Kell Alloimmunization: A Perinatal Approach to a Rare Case. Turk J Haematol. 2012;29(1):72-5.

28. Bek SG, Eren N, Uzay A, Bakirdogen S. Rh (D) alloimmunization treated by double filtration plasmapheresis. Transfus Apher Sci. 2019;58(1):83-6.

29. Bennardello F, Curciarello G. Survey on the prevention and incidence of haemolytic disease of the newborn in Italy. Blood Transfus. 2013;11(4):518-27.

30. Bi SH, Jiang LL, Dai LY, Zheng H, Zhang J, Wang LL, et al. Rh-incompatible hemolytic disease of the newborn in Hefei. World J Clin Cases. 2019;7(20):3202-7.

31. Brumbaugh JE, Morgan S, Beck JC, Zantek N, Kearney S, Bendel CM, et al. Blueberry muffin rash, hyperbilirubinemia, and hypoglycemia: a case of hemolytic disease of the fetus and newborn due to anti-Kp(a). J Perinatol. 2011;31(5):373-6.

32. Chatziantoniou V, Heeney N, Maggs T, Rozette C, Fountain C, Watts T, et al. A descriptive single-centre experience of the management and outcome of maternal alloantibodies in pregnancy. Transfus Med. 2017;27(4):275-85.

33. Colpo A, Tison T, Gervasi MT, Vio C, Vicarioto M, De Silvestro G, et al. Personalized treatment with immunoadsorption and intravenous immunoglobulin in a case of severe Rh alloimmunization during pregnancy unresponsive to plasma - exchange. Transfus Apher Sci. 2017;56(3):480-3.

34. de Assunção RA, Liao AW, Brizot Mde L, Francisco RP, Zugaib M. Changes in fetal myocardial performance index following intravascular transfusion: preliminary report. J Matern Fetal Neonatal Med. 2016;29(16):2697-702.

35. Gottvall T, Filbey D. Alloimmunization in pregnancy during the years 1992-2005 in the central west region of Sweden. Acta Obstet Gynecol Scand. 2008;87(8):843-8.

36. Gudlaugsson B, Hjartardottir H, Svansdottir G, Gudmundsdottir G, Kjartansson S, Jonsson T, et al. Rhesus D alloimmunization in pregnancy from 1996 to 2015 in Iceland: a nation-wide population study prior to routine antenatal anti-D prophylaxis. Transfusion. 2020;60(1):175-83.

37. Haider M, Memon S, Tariq F, Fatima S, Hameed A. Rhesus Isoimmunization: Late-onset Hemolytic Disease of the Newborn Without Jaundice. Cureus. 2020;12(1):e6559.

38. Harper DC, Swingle HM, Weiner CP, Bonthius DJ, Aylward GP, Widness JA. Long-term neurodevelopmental outcome and brain volume after treatment for hydrops fetalis by in utero intravascular transfusion. Am J Obstet Gynecol. 2006;195(1):192-200.

39. Hassan MZ, Iberahim S, Abdul Rahman WSW, Zulkafli Z, Bahar R, Ramli M, et al. Severe anti-D haemolytic disease of fetal and newborn in rhesus D negative primigravida. Malays J Pathol. 2019;41(1):55-8.

40. Karagol BS, Zenciroglu A, Okumus N, Karadag N, Dursun A, Hakan N. Hemolytic disease of the newborn caused by irregular blood subgroup (Kell, C, c, E, and e) incompatibilities: report of 106 cases at a tertiary-care centre. Am J Perinatol. 2012;29(6):449-54.

41. Kriplani A, Malhotra Singh B, Mandal K. Fetal intravenous immunoglobulin therapy in rhesus hemolytic disease. Gynecol Obstet Invest. 2007;63(3):176-80.

42. Lakhwani S, Machado P, Pecos P, Coloma M, Rebollo S, Raya JM. Kell hemolytic disease of the fetus. Combination treatment with plasmapheresis and intrauterine blood transfusion. Transfus Apher Sci. 2011;45(1):9-11.

43. Levy-Zauberman Y, Mailloux A, Kane A, Castaigne V, Cortey A, Carbonne B. Massive fetomaternal hemorrhage secondary to intrauterine intravascular transfusion. Obstet Gynecol. 2011;118(2 Pt 2):439-42.

44. Lieberman L, Callum J, Cohen R, Cserti-Gazdewich C, Ladhani NNN, Buckstein J, et al. Impact of red blood cell alloimmunization on fetal and neonatal outcomes: A single center cohort study. Transfusion. 2020;60(11):2537-46.

45. Manoura A, Korakaki E, Hatzidaki E, Saitakis E, Maraka S, Papamastoraki I, et al. Use of recombinant erythropoietin for the management of severe hemolytic disease of the newborn of a K0 phenotype mother. Pediatr Hematol Oncol. 2007;24(1):69-73.

46. Matijevic R, Grgic O, Klobucar A, Miskovic B. Diagnosis and management of Rh alloimmunization. Fetal Diagn Ther. 2005;20(5):393-401.

47. Mayer B, Hinkson L, Hillebrand W, Henrich W, Salama A. Efficacy of Antenatal Intravenous Immunoglobulin Treatment in Pregnancies at High Risk due to Alloimmunization to Red Blood Cells. Transfus Med Hemother. 2018;45(6):429-36.

48. Meraj B, Mobusher I, Waheed S, Waseem M, Rashid Y. Role of intrauterine blood transfusions in management of Rh-isoimmunized pregnancies. Pakistan J Medical Health Sci. 2015;9:318-21.

49. Navarro M, Negre S, Matoses ML, Golombek SG, Vento M. Necrotizing enterocolitis following the use of intravenous immunoglobulin for haemolytic disease of the newborn. Acta Paediatr. 2009;98(7):1214-7.

50. Nwogu LC, Moise KJ, Jr., Klein KL, Tint H, Castillo B, Bai Y. Successful management of severe red blood cell alloimmunization in pregnancy with a combination of therapeutic plasma exchange, intravenous immune globulin, and intrauterine transfusion. Transfusion. 2018;58(3):677-84.

51. Palfi M, Hildén JO, Matthiesen L, Selbing A, Berlin G. A case of severe Rh (D) alloimmunization treated by intensive plasma exchange and high-dose intravenous immunoglobulin. Transfus Apher Sci. 2006;35(2):131-6.

52. Phung TV, Houfflin-Debarge V, Ramdane N, Ghesquière L, Delsalle A, Coulon C, et al. Maternal red blood cell alloimmunization requiring intrauterine transfusion: a comparative study on management and outcome depending on the type of antibody. Transfusion. 2018;58(5):1199-205.

53. Rahimi-Sharbaf F, Niromanesh S, Talebzadeh Z, Kaveh M, Nayary F. Rh alloimmunization and term delivery. Arch Iran Med. 2007;10(1):111-3.

54. Rath ME, Smits-Wintjens VE, Lindenburg IT, Brand A, van Kamp IL, Oepkes D, et al. Exchange transfusions and top-up transfusions in neonates with Kell haemolytic disease compared to Rh D haemolytic disease. Vox Sang. 2011;100(3):312-6.

55. Rath ME, Smits-Wintjens VE, Lindenburg IT, Folman CC, Brand A, van Kamp IL, et al. Postnatal outcome in neonates with severe Rhesus c compared to rhesus D hemolytic disease. Transfusion. 2013;53(7):1580-5.

56. Ree IMC, de Haas M, Middelburg RA, Zwiers C, Oepkes D, van der Bom JG, et al. Predicting anaemia and transfusion dependency in severe alloimmune haemolytic disease of the fetus and newborn in the first 3 months after birth. Br J Haematol. 2019;186(4):565-73.

57. Ree IMC, Lopriore E, Zwiers C, Böhringer S, Janssen MWM, Oepkes D, et al. Suppression of compensatory erythropoiesis in hemolytic disease of the fetus and newborn due to intrauterine transfusions. Am J Obstet Gynecol. 2020;223(1):119.e1-.e10.

58. Ruma MS, Moise KJ, Jr., Kim E, Murtha AP, Prutsman WJ, Hassan SS, et al. Combined plasmapheresis and intravenous immune globulin for the treatment of severe maternal red cell alloimmunization. Am J Obstet Gynecol. 2007;196(2):138.e1-6.

59. Sainio S, Nupponen I, Kuosmanen M, Aitokallio-Tallberg A, Ekholm E, Halmesmäki E, et al. Diagnosis and treatment of severe hemolytic disease of the fetus and newborn: a 10-year nationwide retrospective study. Acta Obstet Gynecol Scand. 2015;94(4):383-90.

60. Santos MC, Sá C, Gomes SC, Jr., Camacho LA, Moreira ME. The efficacy of the use of intravenous human immunoglobulin in Brazilian newborns with rhesus hemolytic disease: a randomized double-blind trial. Transfusion. 2013;53(4):777-82.

61. Şavkli A, Çetin BA, Acar Z, Özköse Z, Behram M, Çaypinar SS, et al. Perinatal outcomes of intrauterine transfusion for foetal anaemia due to red blood cell alloimmunisation. J Obstet Gynaecol. 2020;40(5):649-53.

62. Simonazzi G, Bernabini D, Curti A, Bisulli M, Pilu G, Brill CB, et al. Fetal cerebellar damage in fetuses with severe anemia undergoing intrauterine transfusions. J Matern Fetal Neonatal Med. 2016;29(3):389-92.

63. Temel Yüksel İ, Acar D, Turhan U, Aslan Çetİn B, Köroğlu N, Şenol G, et al. Assessment of fetal right ventricular myocardial performance index changes following intrauterine transfusion. J Matern Fetal Neonatal Med. 2021;34(18):3046-9.

64. Tiblad E, Kublickas M, Ajne G, Bui TH, Ek S, Karlsson A, et al. Procedure-related complications and perinatal outcome after intrauterine transfusions in red cell alloimmunization in Stockholm. Fetal Diagn Ther. 2011;30(4):266-73.

65. Walsh CA, Russell N, McAuliffe FM, Higgins S, Mahony R, Carroll S, et al. Relationship between maternal antibody type and antenatal course following intrauterine transfusion for red cell alloimmunisation. Eur J Obstet Gynecol Reprod Biol. 2013;171(2):235-9.

66. Xu W. A case of severe Rh (D) alloimmunization pregnant woman delivery an infant with limited treatment. Transfus Apher Sci. 2013;49(2):168-70.
